# Prediction of novel biomarkers for gastric intestinal metaplasia and gastric adenocarcinoma using bioinformatics analysis

**DOI:** 10.1016/j.heliyon.2024.e30253

**Published:** 2024-04-25

**Authors:** Mohammad Reza Eskandarion, Sharareh Eskandarieh, Abbas Shakoori Farahani, Habibollah Mahmoodzadeh, Farhad Shahi, Mohammad Ali Oghabian, Reza Shirkoohi

**Affiliations:** a Cancer Research Center, Cancer Institute, IKHC, Tehran University of Medical Sciences, Tehran, Iran; b Multiple Sclerosis Research Center, Neuroscience Institute, Tehran University of Medical Sciences, Tehran, Iran; c Medical Genetics Ward, IKHC Hospital Complex, Tehran University of Medical Sciences, Tehran, Iran; d Department of Surgery, Cancer Research Center, Cancer Institute, IKHC, Tehran University of Medical Sciences, Tehran, Iran; e Department of Medical Oncology, Cancer Research Center, Cancer Institute, IKHC, Tehran University of Medical Sciences, Tehran, Iran; f Medical Physics Department, Faculty of Medicine, Tehran University of Medical Sciences, Tehran, Iran

**Keywords:** Gastric cancer, Intestinal metaplasia, Differentially expressed genes, Bioinformatics analysis, MicroRNA, TF

## Abstract

**Background & aim:**

The histologic and molecular changes from intestinal metaplasia (IM) to gastric cancer (GC) have not been fully characterized. The present study sought to identify potential alterations in signaling pathways in IM and GC to predict disease progression; these alterations can be considered therapeutic targets.

**Materials & methods:**

Seven gene expression profiles were selected from the GEO database. Discriminate differentially expressed genes (DEGs) were analyzed by EnrichR. The STRING database, Cytoscape, Gene Expression Profiling Interactive Analysis (GEPIA), cBioPortal, NetworkAnalyst, MirWalk database, OncomiR, and bipartite miRNA‒mRNA correlation network was used for downstream analyses of selected module genes.

**Results:**

Analyses revealed that extracellular matrix-receptor interactions (ITGB1, COL1A1, COL1A2, COL4A1, FN1, COL6A3, and THBS2) in GC and PPAR signaling pathway interactions (FABP1, APOC3, APOA1, HMGCS2, and PPARA and PCK1) in IM may play key roles in both the carcinogenesis and progression of underlying GC from intestinal metaplasia. IM enrichment indicated that this is closely related to digestion and absorption. The TF-hub gene regulatory network revealed that AR, TCF4, SALL4, and ESR1 were more important for hub gene expression. It was revealed that the development and prediction of GC may be affected by hsa-miR-29. It was found that PTGR1, C1orf115, CRYL1, ALDOB, and SULT1B1 were downregulated in GC and upregulated in IM. Therefore, they might have tumor suppressor activity in GC progression.

**Conclusion:**

New potential biomarkers and pathways involved in GC and IM were identified that are important for the transformation of GC from IM to adenocarcinoma and can be therapeutic targets for GC.

## Highlights

• ECM interaction in GC and PPAR signaling pathway interaction in intestinal metaplasia may play key roles in the progression of GC.
• COL1A2, COL4A1, and COL6A3 were significantly correlated with overexpression, poor prognosis, and the highest mutation rates in GC.
• FABP1, APOC3, APOA1, HMGCS2, PPARA, and PCK1 are important biomarkers that have received little attention in IM.
• Developing and forecasting the GC via regulating the hub genes may be affected by hsa-miR-29.

## 1 Introduction

Given that gastric cancer (GC) is the fifth most prevalent cancer (5.7 %) and the second most common cause of cancer-related death (8.2 %), it is estimated that nearly ninety percent of GC cases are adenocarcinomas (AC) [1]. Patients with primary GC undergo endoscopy or are treated with gastrectomy with lymphadenectomy, and systemic cancer therapy is regarded as a standard treatment for patients suffering from nonremovable or recurrent GC [2]. Moreover, compared with conventional diagnostic tools, advanced endoscopic methods are considered more reliable tools for detecting GC. However, this technique is limited due to its invasiveness and cost concerns [3]. Serum levels of tumor markers, such as carcinoembryonic antigen (CEA), CA-125, and CA-19-9 are also commonly used for the management of GC patients. However, these methods are not sufficient to detect the disease and determine the prognosis of patients with GC [4]. It is therefore necessary to develop novel diagnostic techniques. Identifying new biomarkers is helpful for designing molecular methods for the early diagnosis and monitoring of patients. Recently, many studies have been conducted in this field both at the bioinformatics level and at the genomic level, and many biomarkers have been identified. However, many of the identified biomarkers have not been proven experimentally, so conducting comprehensive studies in this field is necessary. First, the path of stomach cancer formation should be reviewed. IM is considered a precancerous condition of gastric adenocarcinoma (GAC) related to an increased risk of developing GC. IM is considered a *trans*-differentiation process progressing from the gastric epithelium to an intestinal type, both of which are mostly induced by *H. pylori* infection and the expression of the homeobox CDX1 and CDX2 genes. It is a protective reaction to inflammation, but IM also results in an increased risk of neoplastic transformation [5]. Patients suffering from IM are at a greater risk of GC, and the annual incidence of GC is 0.13%–0.25 % for these patients [6]. Previous studies have suggested that based on histology, IM can be categorized into two subtypes: ‘‘low-risk’’ complete (CIM, type I, small intestine) and ‘‘high-risk’’ (IM, types II and III, colonic) [7]. According to epidemiological studies, the progression rate of incomplete-type IM to GC is greater than that of complete-type IM to GC [8]. However, the associations between histologic and molecular changes from IM to GC are still controversial, and the genes and pathways involved in this progression are not known. Therefore, it is important to identify genes and molecular processes involved in this transition because they could reveal hub genes involved in tumor progression and potential novel biomarkers as well as therapeutic targets.

Therefore, the current research aimed to identify important genes and signaling pathways involved in the development of GC and determine important molecular markers in this pathway by using bioinformatics and designing a comprehensive study.

## 2 Materials and methods

### 2.1 Data collection

There are many datasets in the Gene Expression Omnibus (GEO) database, and only some of these databases can be used for identifying hub genes involved in tumor progression from IM to GC. Therefore, five selection criteria were employed.

1. All the samples should be for Homo sapiens.
2. Data can be analyzed with GEO2R.
3. The number of samples should be more than six.
4. In each dataset, normal subjects and patient groups were compared.
5. In the IM group, patients with GIM-GC, patients with GIM without progression to GC (GIM-NoGC), and healthy controls were included.

A total of six gene expression profiles (GSE54129, GSE79973, GSE103236, GSE33651, GSE19826, and GSE118916), including information on GC and one gene expression profile (GSE78523) on intestinal metaplasia, were used to identify the effective molecular targets and pathways and GSE93415 on platform GPL19071, related to the miRNA expression level of 20 GC samples and 20 healthy samples, which were selected from the NCBI-GEO database (https://www.ncbi.nlm.nih.gov/geo/) [9]. GSE54129, GSE19826, and GSE79973 are based on the GPL570 platform, and GSE103236, GSE118916, GSE33651, and GSE78523 are based on the GPL4133, GPL15207, GPL2895, and GPL18990 platforms, respectively. Using other datasets for the validation of key genes in this study, the GSE13911, GSE191275, GSE65801, and GSE174237 datasets were used from the GPL570, GPL20301, GPL14550, and GPL16791 platforms, respectively.

### 2.2 Data processing of DEGs

The differentially expressed genes (DEGs) between GC and normal samples and between IM and normal samples were detected by using GEO2R online analysis tools (https://www.ncbi.nlm.nih.gov/geo/geo2r/) with adjusted P*-*values<0.01 and |log2FC|>1 as the cutoff criteria and by dividing the DEGs into two categories with upregulated and downregulated gene expression scores, and the statistical significance of these DEGs was determined. Common data between gene expression profiles were defined by web-based Venn diagram software (http://bioinformatics.psb.ugent.be/cgi-bin/liste/Venn/calculate_venn.htpl). Normalization and data processing were performed with the GEO2R online analysis tool, which applies quantile normalization to the expression data.

### 2.3 Enrichment analysis

Both GO (Gene Ontology) and KEGG (Kyoto Encyclopedia of Genes and Genomes) were employed to identify possible signaling pathways involved in analyzing BP, MF, and CC, which represent biological process, molecular function, and cellular component, respectively. Furthermore, functional analysis of the selected genes was performed using the Enrichr web server [[10], [11], [12], [13], [14]] and the Database for Annotation, Visualization, and Integrated Discovery (DAVID) (https://david-d.ncifcrf.gov). Additionally, an adjusted P-value<0.05 was regarded as the cutoff criterion [15,16]. The functional annotation of the genes involved in the modules was performed by using Enrichr. All the hub genes related to GC and IM were reanalyzed by KEGG pathway enrichment.

### 2.4 Protein‒protein interaction (PPI) network and module analysis

The Search Tool for the Retrieval of Interacting Genes (STRING) (https://string-db.org/) was used to assess the protein–protein interaction (PPI) network with an adjusted P-value>0.4, which was set as the cutoff criterion [17,18]. The cluster analysis between DEGs was performed by using Cytoscape version 3.7.2 [19]. The Molecular Complex Detection (MCODE) plugin of Cytoscape was utilized to identify the modules involved in the PPI networks with a degree cutoff = 2, node score cutoff = 0.2, maximum depth = 100, and k-score = 2. The CytoHubba plugin of the Cytoscape program was used to identify the hub genes and evaluate the degree, maximum neighborhood component (MNC), maximal clique centrality (MCC), and edge percolated component (EPC) of the resulting network [20].

### 2.5 Survival analysis of hub genes

The overall survival of the core genes was evaluated by the Kaplan‒Meier plotter [21,22]. The expression levels associated with the core genes were determined by Gene Expression Profiling Interactive Analysis (GEPIA) [23]. GEPIA, as a web-based tool, can achieve characteristic functionalities based on the TCGA and GTEx databases. The hazard ratio (HR) with 95 % confidence interval (95 % CI) and the P-value for the log-rank test were calculated and plotted.

### 2.6 Mutation analysis of hub genes

C-BioPortal (http://cbioportal.org) [24,25] was used for mutation analysis of the hub genes involved in GC and IM. C-BioPortal is an online tool for obtaining data on GC from whole-genome sequencing of 147 GC tumors and matched normal tissues. Courtesy of OncoSG [26], whole-genome sequencing of 100 GC tumor-normal pairs, was performed at the University of Hong Kong and Pfizer [27]. Whole-genome sequencing was performed on 478 samples from TCGA Stomach Adenocarcinoma. The source data used were from GDAC Firehose, previously known as TCGA Provisional [28]. Whole-exome sequencing of 30 diffuse-type gastric adenocarcinoma samples was performed (with matched normal samples) from the University of Tokyo [29], and exome sequencing of 22 GC samples was carried out with matched normal samples [30].

### 2.7 Gene regulatory network analysis

NetworkAnalyst (https://www.networkanalyst.ca/) [31,32] was used to construct a regulatory network of the hub genes and TFs (transcription factors), after which the TFs with adjusted P-value<0.05 in the ChEA were visualized via Cytoscape.

### 2.8 Potential miRNA‒mRNA interactions

Differentially expressed miRNAs (DEmiRs) between GC samples and normal tissues in GSE93415 were identified using GEO2R online analysis tools with P-value<0.05, adjusted P*-*value<0.01, and |log2FC|>1 as the cutoff criteria. The miRWalk database was then used to identify the target genes of the DEmiRs. The common genes between the target genes and the chosen module genes were identified using a Venn diagram. Finally, the Cytoscape program was used to construct and assess a bipartite miRNA‒mRNA correlation network [33]. OncomiR (http://www.oncomir.org/) [34] was used to explore microRNA expression in normal tissues compared with cancer tissues of different stages and grades.

## 3 Results

### 3.1 Identification of DEGs

**Fig. 1:**
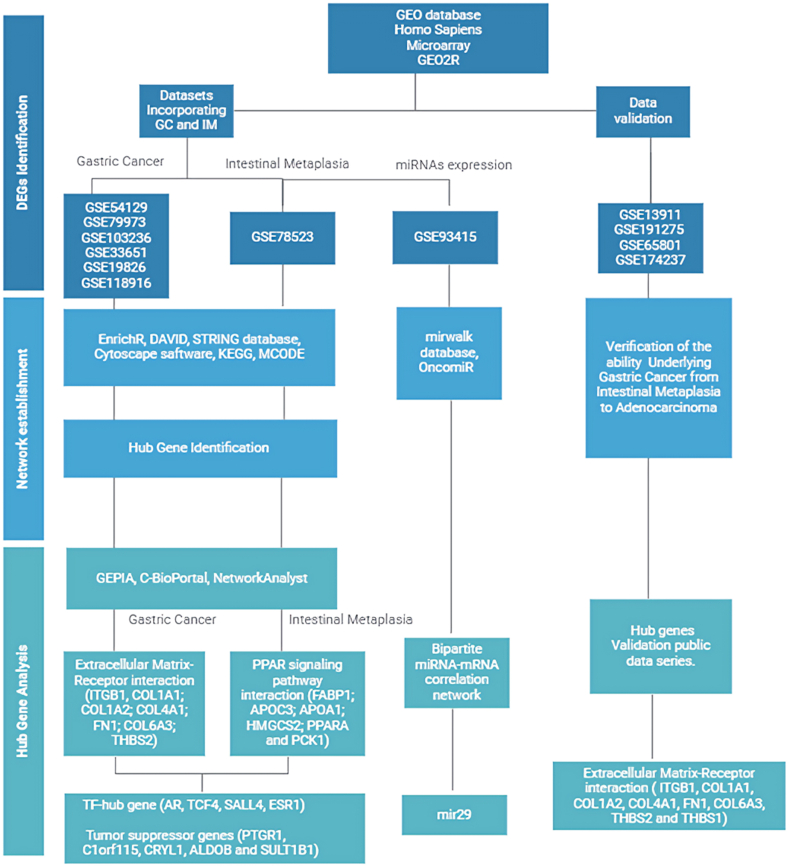
A flow diagram of the study. GC: Gastric Cancer; IM: Intestinal Metaplasia; GEO: Gene Expression Omnibus; DEG: Differentially Expressed Gene; DAVID: Database for Annotation, Visualization, and Integrated Discovery; STRING: Search Tool for the Retrieval of Interacting Genes; KEGG: Kyoto Encyclopedia of Genes and Genomes; MCODE: Molecular Complex Detection; GEPIA: Gene Expression Profiling Interactive Analysis.

As shown in Fig. 1, the flow diagram of this study demonstrates that a total of six gene expression datasets, GSE54129, GSE79973, GSE103236, GSE33651, GSE19826, and GSE118916, were selected from the GEO-NCBI for GC, and one dataset, GSE78523, was selected for intestinal metaplasia. The GSE93415 dataset was related to the miRNA expression level, and the GSE13911, GSE191275, GSE65801, and GSE174237 datasets were selected for validation of key genes (Table 1). Adjusted P-values<0.01 and |log2FC|>1 was considered for all analyses. A total of 7795 DEGs were detected for gene expression, including 54 upregulated genes and 28 downregulated genes for GSE103236, 851 upregulated genes and 822 downregulated genes for GSE118916, 86 upregulated genes and 917 downregulated genes for GSE19826, 76 upregulated genes and 622 downregulated genes for GSE33651, 1859 upregulated genes and 2078 downregulated genes for GSE54129, and 145 upregulated genes and 257 downregulated genes for GSE79973. A total of 221 upregulated DEmiRs and 223 downregulated DEmiRs were identified in GSE93415. A Venn diagram displayed that 98 upregulated DEGs and 126 downregulated DEGs revealed overlapping among the three datasets (Table 2 and Supplementary Figs. 1A and B). A total of 45687 (23385 upregulated and 22302 downregulated) DEGs in the GSE13911, GSE191275, GSE65801, and GSE174237 datasets were identified as validation data. The common DEGs between all datasets for GC and IM are shown in Table 3 and Supplementary Fig. 1C.

**Table 1:** The details of the GEO datasets.

ID	Data type	Tissue	Platform	Tumor	Normal
GSE79973	mRNA	GC	GPL570	10	10
GSE103236	mRNA	GC	GPL4133	10	9
GSE33651	mRNA	GC	GPL2895	40	12
GSE19826	mRNA	GC	GPL570	12	15
GSE118916	mRNA	GC	GPL15207	15	15
GSE54129	mRNA	GC	GPL570	111	21
GSE93415	miRNA	GC	GPL19071	20	20
GSE13911	mRNA (for validation)	GC	GPL570	38	31
GSE191275	mRNA (for validation)	GC	GPL20301	10	10
GSE65801	mRNA (for validation)	GC	GLP14550	32	32
GSE174237	mRNA (for validation)	GC	GLP16791	6	4
GSE78523	mRNA	IM	GPL18990	14	15

**Table 2:** Venn diagram results from six GEO datasets for GC and one dataset for IM.

	DEGs	Gene names
GC	Upregulated	FSTL1 SFRP4 CDH3 SPARC KIF26B ENO1 COL5A1 PDPN SDS LAMA4 WASF1 FNDC1 PMEPA1 COL3A1 COL4A1 TUBB6 VCAN LRP8 ADAM12 GLS CAP2 TNFRSF11B ACTN1 CEMIP LUM FAM83D COL18A1 TRIO CTHRC1 IGF2BP3 PRKCDBP BCAT1 TMEM158 CDH11 CHN1 SULF1 FRMD6 THBS1 SERPINH1 HMCN1 ITGB1 FKBP10 AEBP1 COL5A2 FBN1 FAP BMP1 TIMP1 EPHB2 IGFBP7 RARRES1 THBS2 RAB31 THY1 TNFSF4 BGN INHBA PTPN12 LCP1 ANTXR1 COL1A2 NID2 CORO1C COL6A3 TNFRSF10B SPHK1 SPP1 CMTM3 MFAP2 CRISPLD1 COL1A1 HTRA1 ALDH1B1 APOC1 WISP1 COL8A1 SNX10 DPYSL3 TREM2 ANOS1 COL12A1 CLDN1 NME7 TIMP2 NOX4 FN1 TEAD4 OLFML2B LGALS1 LOXL1 SRPX2 CALD1 SALL4 ECT2 COL10A1 PRRX1 ASPN NDE1
	Downregulated	MAL ESRRG RAB11FIP4 PPID IGSF3 C1orf115 GPAT3 EPB41L4B GATA6 F2RL1 GKN1 ALAD STX19 PKIB GPRC5C PBLD PXMP2 DGKD CYSTM1 CYP3A5 NEDD4L SVIP SH3BGRL2 DOPEY2 CYP2C9 SGK2 GCNT2 SCNN1B ZBTB7C CWH43 PYROXD1 PRELID2 GRAMD1C LYPD6B BLNK ATP11B TSPAN12 SLC22A23 HRASLS2 GIF PPP1R36 PLEKHH1 SMPD3 SMIM6 TST MYZAP STS SMIM5 B3GNT6 AKR1C1 CRYL1 BTD SCNN1G CYB5A PSAPL1 ABCA5 KCNJ16 SLC26A7 ZNF57 LNX1 GKN2 BCL2L14 FAM20A PROM2 AKR1B10 PGRMC2 ATP4A MAGI3 FGD4 FOLR1 AQP4 CAPN9 CPA2 LRRC66 ACKR4ADH7 SULT2A1 PTER SMPDL3A C1orf210 CCKBR ANG HPGD OASL GDPD1 SLC7A8 TMPRSS2 ENTPD5 CAPN13 MYRF EPN3 UBL3 ETFDH NQO1 CYP2C18 ALDH3A1 PTGR1 HADH RDH12 ELOVL6 SLC9A1 ATP4B SLC26A9 SLC41A2 PRLR GOLGA2P10 SSTR1 PPFIBP2 PLLP PLPP5 HIPK2 ALDOB KCNJ15 SULT1B1 RASSF6 ATP8B1 TRIM50 CBR1
IM	Upregulated	VDR CD44 ANXA2 CDH3 GSKIP XPNPEP2 RGS2 XDH ECHS1 TNIK SLC22A18AS ATP5F1 VIL1 PHGR1 SLC25A3 FAM3C GGT1 GSTA1 BRI3BP ITPK1 HSD17B2 SULT1E1 TMEM139 WNK2 HAS3 ABCG2 HMOX1 EPPK1 ARHGAP32 SLC39A4 HMGCS2 FAM134B ERVK-7 TMEM45B SLC2A5 SERPINB5 REEP6 ZG16 SLC25A37 EXT1 CLDN15C20orf24 AATK GDA CEACAM6 ITLN1 HEPH EMB MS4A10 ATP2C2 PPARA GK AFAP1-AS1 UGT1A1 CHP2 GIPC2 HOXA13C15orf41 BCMO1 STRADB PTGDS EPT1 DHRS11 GPR153 ARL4A FAM83B HSD11B2 LYN DUSP6 CDCA3 LUZP1 PAPSS2 CLDN4 PFKFB2 SLC22A18 MYO1A CES3 PRSS2 YBX2 SLCO2B1 CREB3L3 UGT2B7 CD55 A1CF AC009133.14 OLFM4 CFI REG4 DMXL2 CALML4 FABP1 MOGAT3 IGKC CES2 SLC15A1 TTLL6 STARD5 ANXA13 CLDN3 TMEM150B DPP4 APOC3 APOBEC1 DMBT1 ACOT11 SOD1 GPR128 LPGAT1 MUC3A PLA2G2A SEC16B NPY6R TM4SF4 MAOA CASP10 SRI MBNL1 SIRPA CEACAM20 SGPL1 ATP5G3 VNN1 TRIM31 NEUROG3 GRIN2D HSPD1 SLC1A1 LCOR LGALS3 PTGR1 FUT3 SERPINB6 SLC6A19 MIER3 NAT2 TRABD2A SLC26A3 SLC7A9 KRT20 LOC100124692 MYO15B HTATIP2 QPRT VIMP ETHE1 PDZD3 HSD17B11 GPD1 PCK2 PAG1 LINC00483 MUC17 EVI2B AKAP9 ASS1 GSTM4 GABRP PDE5A SLC30A10 MYRFL B3GALT5 WDR72 CEBPG HECTD3 C19orf77 HHLA2 SLC20A2 CMAS CHST6 SPINK4 SMLR1 APOB MICAL3 SLC6A8 PIGR ACOT7 ETS2 TBX3 ACAA1 FUT6 AHCYL2 SEMA6D SIPA1L2 GIP CDX2 HOXB13 PLD1 HOXB6 CEACAM18 CNIH HNF4A TNFRSF10B MACC1 TP53INP2 SERPINA1 ANTXR2 DDC TNFRSF11A ANPEP MID1 SLC9A3R1 CDHR5 CYP4F3 SLC39A5 CFTR MTTP HADHB CEACAM7 ALPI ADIRF CLDN7 GLRX SLC3A1 MLN ABCG5 TFAP2A MGAT4A SLC46A1 FCRL5 NR1H4 ELOVL7 KRT7 S100A10 TPMT KIF13A ATP1A1 SOX6 TFPI HTR1D C11orf86 CDH17 CLCA1 DGKA FAM84A RAB30 ATP7B SDHC PPP1R1B SLC19A3 EFNA2 TRIM40 GLS HOXB5 PSMD1 RNF186 PRELID1 MUC12 NHSL1 TMEM41A ABP1 MYOF TLDC2 KIAA1161 DAK C1QBP MLEC MUC2 MSLN PDK1 SLC13A2 TM4SF20 DEFA6 KIAA1211 PLK1S1 PLS1 SLC35A3 SLC51B NMUR2 SLC38A1 IL32 TOMM40L MMD CCL14 ADAMTSL5 TMEM253 ABCC2 METTL7B PLEKHS1 PGM1 GCG FAT1 UGT1A8 CPS1 GCNT3 CHST5 SIM2 LDHA SLC35F2 CCND2 LOC101060198 GBA3 MARVELD3 ZNF488C1orf115 SLC46A3 CASP1 PLIN3 MPP1 SLC5A1 ABCG8 CRYL1 MLK7-AS1 BTNL3 HADHA PEX26 LGALS4 PON2 KLK1 PCK1 KBTBD11 GALC CLDN1 CCL25 GPRC5A ANK3 UBE2J1 CHN2 ABHD14A-ACY1 SLC27A2 CLRN3 MUC13 ARSE NT5E C10orf112 OVOL1 CCL15 CIDEC SLC35G1 ADH6 GALM HNF4G PRR15L APOA4 SLC17A4 HEPACAM2 SIAE SLC30A4 UGT2A3 ODF2L TINAG ABCC13 ACE2 VIPR1 DGKQ USP2 TMEM106C SLC5A9 CEACAM1 ALDOB F3 FAM160A2 CTSZ NR1I2 SSUH2 TRIB1 APOA1 NPNT DEFA5 ACSL5 C2orf88 HKDC1 PRAP1 CDKN2B DQX1 RETNLB PPP1R14D GPA33 SLC4A7 LOC100127947 CIDEB MME TFRC CAMK2N1 TM6SF2 CA1 HOXB7 AGMAT SLC37A1 NAT1 CYBRD1 REG3A MTCH2 MUC4 ONECUT2 ACAA2 FABP2 NRG1 S100A16 CDA TRIM15 ESPN SULT1B1 PPARGC1B EPHB2 ATP1B3 BAIAP2L2 PLOD2 FAM3D PI3 TMEM168 SLC4A4 DLEU1 BHLHE41 AIMP2 TFF3 ACHE MEP1B PEBP1 ABHD17C MYO7B TRIM36 MTMR11 TDP2 ATP10B PANK3 FAM211A TTC38 LAPTM4B GLTPD2 MOGAT2 SAMD5 SI ARHGAP27 CYP3A4 MISP CDKL1 AP001065.2 FLVCR2 UQCRH CCL24 PEPD CARD16 APP SLC51A PARM1 SEMA6A CD68 EPCAM EPHA1 TMPRSS15 EAF2 PRDM1 CDX1 SLC25A15 IDH3A ACY3 FGFBP1 CDHR2 LBR MOB3B TOP1 SUCLG1 IL2RG OAT AGPAT9 MYB SMOC2 CTSC XPR1
	Downregulated	LYPD6B SPINK1 CHGB LL22NC03–75H12.2 ZSCAN18 DPT CGNL1 FUT1 LOC400043 FAM20A SOX21 TCN1 LOC100130533 HOMER2 SLC5A5 CARNS1 GALNT6 B4GALNT3 ARHGEF28 FOXA2 FGA ALDH3A1 FUT9 MUC1 CCKAR GLUL C16orf89 PP7080 PCDHGB5 MAL ERO1LB NKX6-2 MFSD4 SOX21-AS1 FZD8 GPR64 PDE4C SLC29A1 APLP1 CRMP1 DGKD FAM189A2 MICALL1 CSTA SNAP25 PGC KLF2 CA9 FAM159B RMST GPRC5B SST TFF2 MAP7D2 FMOD SCNN1B ZNF662 CES1 CES1P1 CCKBR FLJ42875 PSAPL1 KLK11 GAST TESC KCNJ15 HAP1 TMEM63B CTC-436P18.3 SCGB2A1 PCSK2 SCG5 TSPAN5 CXCL17 PCSK1N GRIP2 EEF1A2

**Table 3:** All common DEGs found between GC and IM.

	Gene names
**GC UP and IM UP**	CDH3 TNFRSF10B GLS CLDN1 EPHB2
**GC DOWN and IM UP**	PTGR1 C1orf115 CRYL1 ALDOB SULT1B1
**GC DOWN and IM DOWN**	LYPD6B FAM20A ALDH3A1 C16orf89 MAL DGKD SCNN1B CCKBR PSAPL1 KCNJ15
**GC UP and IM DOWN**	–

### 3.2 Functional enrichment analysis

Enrichr, which is a comprehensive web server for performing gene set enrichment analysis, was used to analyze functional enrichment. The results obtained from GO analysis demonstrated that the DEGs were considerably enriched in BP, CC, and MF for GC (Supplementary Table 1) and intestinal metaplasia (Supplementary Table 2). Furthermore, enriched KEGG pathways were analyzed to identify the important pathways associated with the DEGs. As indicated in Table 4, KEGG analysis demonstrated that the DEGs for GC were upregulated in the digestion and absorption of protein, interactions between the ECM and receptor, and focal adhesion. Those for GC were downregulated in the secretion of gastric acid, metabolism of xenobiotics by cytochrome P450, and metabolism of glycerolipids. KEGG pathway enrichment analysis of IM revealed that DEGs for IM were upregulated in the bile secretion, digestion and absorption, mineral absorption, the signaling pathway of PPAR, and digestion and absorption protein.

**Table 4:** KEGG in GC and IM.

	Expression	Term	P-value	Gene
GC	**Upregulated**	Protein digestion and absorption	3.02E-12	COL1A1; COL18A1; COL3A1; COL1A2; COL5A1; COL4A1; COL12A1; COL5A2; COL8A1; COL10A1; COL6A3
		ECM-receptor interaction	1.64E-11	ITGB1; COL1A1; COL1A2; COL4A1; LAMA4; SPP1; FN1; COL6A3; THBS2; THBS1
		Focal adhesion	4.20E-09	COL1A1; ITGB1; COL1A2; COL4A1; ACTN1; LAMA4; FN1; SPP1; COL6A3; THBS2; THBS1
		Amoebiasis	6.89E-07	COL1A1; COL3A1; COL1A2; COL4A1; ACTN1; LAMA4; FN1
		Human papillomavirus infection	5.20E-06	COL1A1; ITGB1; COL1A2; COL4A1; LAMA4; FN1; SPP1; COL6A3; THBS2; THBS1
	**Downregulated**	Gastric acid secretion	5.08E-07	ATP4B; ATP4A; CCKBR; KCNJ15; KCNJ16; SLC26A7; SLC9A1
		Metabolism of xenobiotics by cytochrome P450	5.08E-07	CBR1; ALDH3A1; CYP2C9; AKR1C1; ADH7; CYP3A5; SULT2A1
		Retinol metabolism	6.95E-05	CYP2C9; RDH12; ADH7; CYP2C18; CYP3A5
		Glycerolipid metabolism	5.94E-04	PLPP5; DGKD; AKR1B10; GPAT3
		Aldosterone-regulated sodium reabsorption	0.001622	SCNN1G; SCNN1B; NEDD4L
IM	**Upregulated**	Bile secretion	3.83E-11	ABCG8; ABCG5; ABCC2; UGT1A1; NR1H4; ATP1B3; SLC51A; ATP1A1; SLC5A1; SLC51B; CYP3A4; SLC4A4; UGT2A3; UGT1A8; CFTR; UGT2B7; ABCG2
		Fat digestion and absorption	3.74E-09	FABP1; ABCG8; FABP2; ABCG5; MOGAT3; MOGAT2; MTTP; PLA2G2A; APOA1; APOA4; APOB
		Mineral absorption	1.50E-08	SLC46A1; SLC6A19; ATP7B; HEPH; VDR; HMOX1; CYBRD1; ATP1B3; ATP1A1; SLC5A1; SLC26A3; SLC39A4
		Drug metabolism	4.36E-08	GSTM4; CDA; TPMT; UGT1A1; MAOA; CYP3A4; ADH6; NAT1; NAT2; GSTA1; UGT2A3; UGT1A8; XDH; CES2; UGT2B7
		PPAR signaling pathway	1.73E-07	FABP1; FABP2; GK; ACSL5; APOA1; APOC3; HMGCS2; ACAA1; PPARA; PCK1; SLC27A2; PCK2
	**Downregulated**	Gastric acid secretion	2.01E-04	CCKBR; SST; KCNJ15; GAST
		Nitrogen metabolism	0.001868	CA9; GLUL
		Histidine metabolism	0.003134	ALDH3A1; CARNS1
		beta-Alanine metabolism	0.005786	ALDH3A1; CARNS1
		Glycosphingolipid biosynthesis	0.012694	FUT9; FUT1

### 3.3 PPI and modular analysis and the identification of hub genes

A total of 129 differential genes involving 129 nodes and 507 edges in GC and 411 differential genes involving 411 nodes and 1484 edges in IM were identified via the STRING 11.0 database using Cytoscape 3.8.0, and the resulting PPI network was analyzed (Supplementary Fig. 2 (A-F)). The CytoHuuba plugin in Cytoscape software was used to determine the top 20 genes based on the degree, MNC, MCC, and EPC methods. The Venn diagram generated by the three methods revealed 18 hub genes related to GC (Table 5 and Supplementary Fig. 3 (A, B)), all of which were upregulated in GC and IM.

**Table 5:** The top core genes screened in GC and IM based on the degree, MNC, MCC, and EPC.

	Gene Symbol	Gene Description
GC	FN1	Fibronectin 1
	COL1A1	Collagen alpha-1(I)
	COL1A2	Collagen alpha-2(I)
	COL3A1	Collagen alpha-1(III)
	BGN	Biglycan
	COL5A2	Collagen type v
	COL5A1	Collagen alpha-1(V)
	COL4A1	Collagen alpha-1(IV)
	SPARC	secreted protein acidic and rich in cysteine
	THBS2	Thrombospondin-2
	FBN1	Fibrillin-1
	COL6A3	Collagen alpha-3(VI)
	LUM	Lumican
	THBS1	Thrombospondin-1
	COL12A1	Collagen alpha-1 (XII)
	TIMP1	Metalloproteinase inhibitor 1
	ITGB1	Integrin beta-1
	VCAN	Versican core protein
IM	APOB	Apolipoprotein B
	HNF4A	Hepatocyte nuclear factor 4-alpha
	PPARA	Peroxisome proliferator-activated receptor alpha
	FABP1	Fatty acid-binding protein
	NR1H4	Nuclear Receptor Subfamily 1 Group H Member 4
	APOA4	Apolipoprotein A-IV;
	ABCC2	Atp-binding cassette, subfamily c
	HMGCS2	Hydroxymethylglutaryl-CoA synthase
	APOC3	Apolipoprotein C-III
	APOA1	Apolipoprotein A-I
	PCK1	Phosphoenolpyruvate carboxykinase
	ALDOB	Fructose-bisphosphate aldolase

### 3.4 Hub gene enrichment

**Fig. 2:**
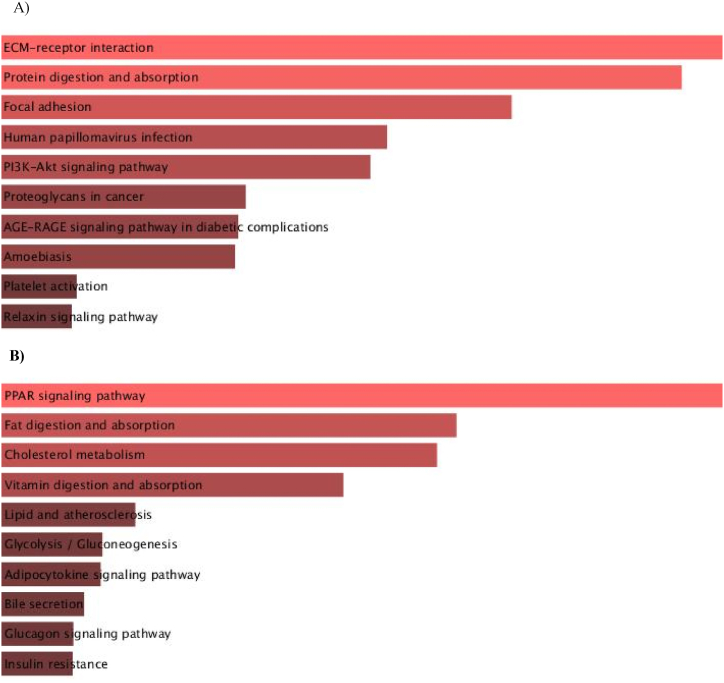
Analysis of the top genes in GC (a) and IM (b) by KEGG pathway enrichment.

All core genes related to GC and IM were reanalyzed by KEGG pathway enrichment. The results obtained from the reanalysis revealed that eight genes (ITGB1, COL1A1, COL1A2, COL4A1, FN1, COL6A3, THBS2, and THBS1) were considerably enriched in the interaction between the extracellular matrix (ECM) and receptor in GC (P < 0.05, Fig. 2A), and six genes (FABP1, APOC3, APOA1, HMGCS2, PPARA, and PCK1) were mostly enriched in the interaction of the PPAR signaling pathway in the IM (P < 0.05, Fig. 2B). Therefore, these top genes observed in GC and IM are hub genes that we need to focus on.

### 3.5 Expression analysis of core genes in GC and IM

**Fig. 3:**
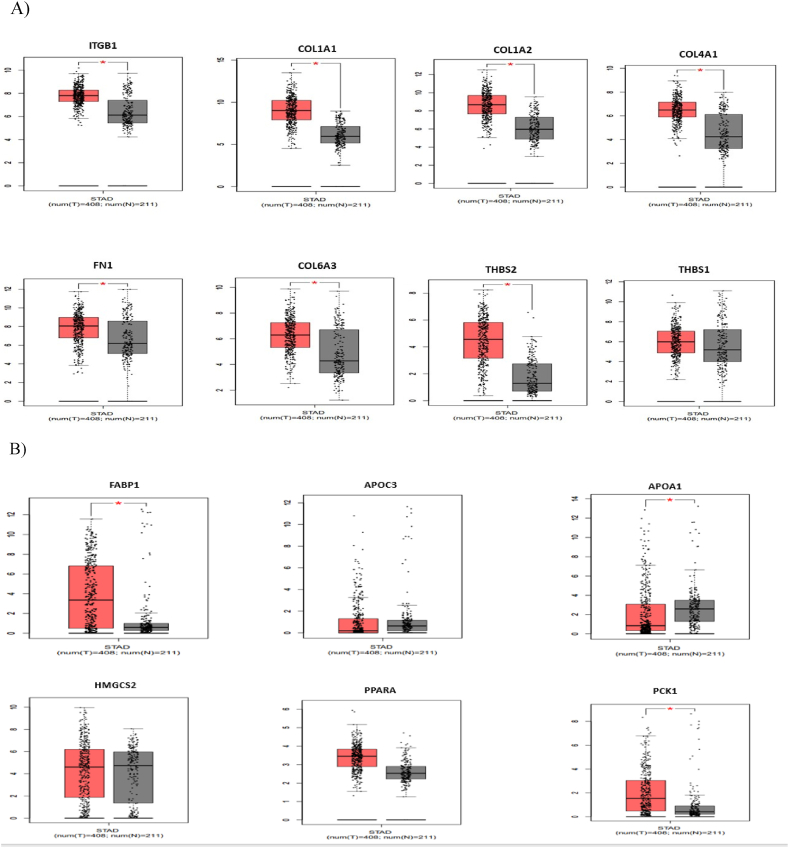
Differentially expressed eight hub genes in GC patients (3A) and six hub genes in IM patients (3B) (red) compared to healthy controls (gray) according to the GEPIA online database (P < 0.05). (For interpretation of the references to colour in this figure legend, the reader is referred to the Web version of this article.)

The expression of the eight hub genes selected from the core gene analysis step was verified by using the GEPIA database. The results demonstrated that all of the genes, except for THBS1, were upregulated in the GC and IM, and the expression levels of the FABP1, PPARA, and PCK1 genes were greater than those in the normal tissues (P < 0.05, Fig. 3A–B).

### 3.6 Prognosis and survival rates of the core genes in GC and IM

**Fig. 4:**
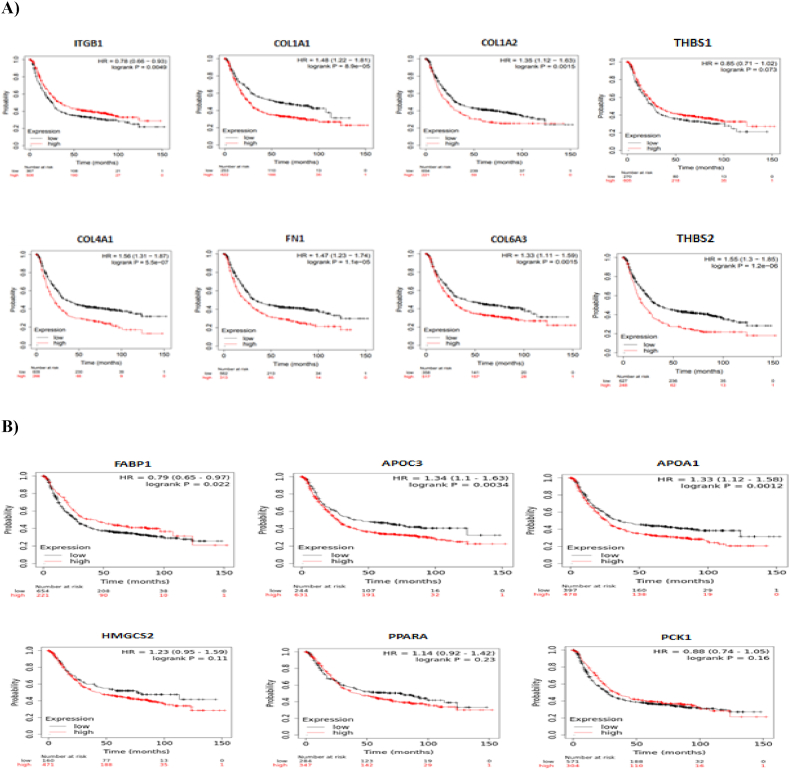
The Kaplan-Meier plotter used to determine the prognostic value of eight core genes in GC (4A) and six hub genes in IM (4B) and significantly related to the survival rate (P < 0.05).

The Kaplan-Meier plotter was used to determine the prognostic value of the top genes related to GC and IM. COL1A1 (P = 8.9e−05), COL1A2 (P = 0.0015), COL4A1 (P = 5.5e−07), FN1 (P = 1.1e−05), COL6A3 (P = 0.0015), and THBS2 (P = 1.2e−06) were significantly associated with poor survival probability in patients with GC, and THBS1 (p = 0.073) was not significantly associated with prognosis and survival rate in patients with GC. Additionally, ITGB1 (P = 0.0049) was demonstrated to be related to favorable overall survival in patients with GC (Fig. 4A). The Kaplan-Meier plotter revealed that FABP1 (P = 0.022), APOC3 (P = 0.0034), and APOA1 (P = 0.0012) were strongly related to the prognosis of IM patients, and HMGCS2 (P = 0.11), PPARA (P = 0.23), and PCK1 (P = 0.16) were not notable markers of the prognosis or survival rate of IM patients (Fig. 4B).

### 3.7 Mutational analysis of hub genes involved in GC and IM

The results obtained from mutational analysis of 777 samples in five studies revealed that mutations were mostly in COL1A2, COL4A1, and COL6A3 (Supplementary Fig. 4A). Mutation analysis of the hub genes involved in IM revealed that PCK1 was the most important gene associated with amplification mutations (Supplementary Fig. 4B).

### 3.8 Gene regulatory network analysis

**Fig. 5:**
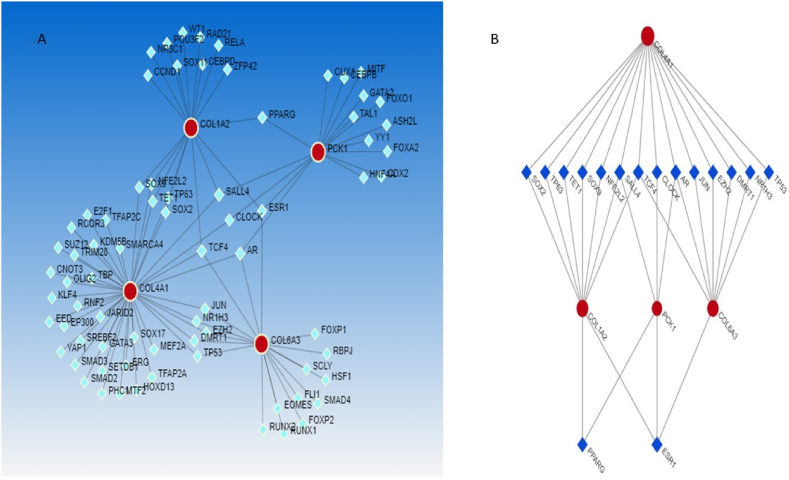
Transcriptional regulatory networks (TRNs) consisting of 78 edges and 66 nodes were constructed for the hub genes (Fig. 5A). Various hub genes were regulated by TFs with a degree ≥2. The gastric tissue filters used are shown in Fig. 5B.

TFs showing an adjusted P-value<0.05 in ChEA via NetworkAnalyst were visualized by using Cytoscape to further understand the regulatory network between TFs and hub genes (Fig. 5A,B and Table 6).

**Table 6:** Transcription factors associated with the hub genes.

Gene	TFs	Total
AR	COL4A1, COL6A3, PCK1	**3**
TCF4	COL4A1, COL1A2, COL6A3	**3**
SALL4	COL4A1, COL1A2, PCK1	**3**
ESR1	COL1A2, COL6A3, PCK1	**3**
NFE2L2	COL4A1, COL1A2,	**2**
TP63	COL4A1, COL1A2	**2**
SOX2	COL4A1, COL1A2	**2**
TP53	COL4A1, COL6A3,	**2**
TET1	COL4A1, COL1A2	**2**
SOX9	COL4A1, COL1A2	**2**
DMRT1	COL4A1, COL6A3,	**2**
EZH2	COL4A1, COL6A3	**2**
JUN	COL4A1, COL6A3,	**2**
CLOCK	COL4A1, PCK1,	**2**
NR1H3	COL4A1, COL6A3,	**2**
PPARG	COL1A2, PCK1	**2**

### 3.9 Bipartite miRNA‒mRNA network analysis

**Fig. 6:**
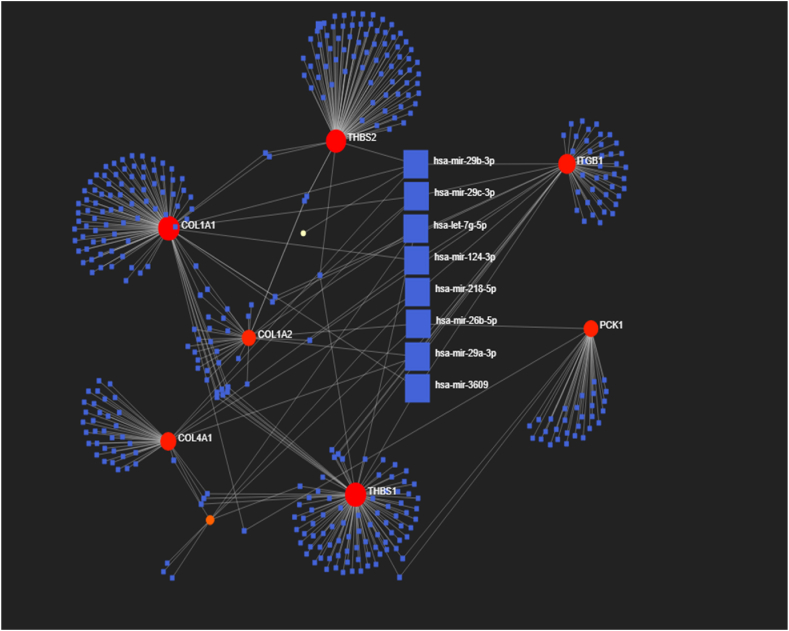
Bipartite miRNA‒mRNA regulatory network of important miRNAs involved in GC progression from IM to adenocarcinoma.

To investigate the role of selected hub genes (ITGB1, COL1A1, COL1A2, COL4A1, FN1, COL6A3, THBS2, THBS1, and PCK1) in the development of IM to adenocarcinoma, possible microRNAs that might interact with these genes via the MirWalk database were predicted, and the miRTarBase filter was considered. Evaluation of common miRNAs between these genes led to the identification of eight miRNAs that are important in IM and GC (Table 7 and Fig. 6). The expression levels of microRNAs in GC tissues and normal tissues were analyzed based on the Oncomine database, revealing that hsa-miR-29b-3p, hsa-miR-29c-3p, hsa-let-7g-5p, hsa-miR-218-5p, and hsa-miR-29a-3p, where tumorigenesis was significantly associated with their expression (Table 8(.

**Table 7:** The miRNAs involved in the hub genes.

miRNA	Gene	Total
hsa-mir-29b-3p	COL4A1,ITGB1,COL1A1,COL6A3,THBS2	5
hsa-mir-29c-3p	COL4A1, ITGB1, COL1A2, COL1A1	4
hsa-let-7g-5p	COL1A2, PCK1, FN1	3
hsa-mir-124-3p	COL4A1, ITGB1, COL1A2	3
hsa-mir-218-5p	COL4A1, ITGB1, FN1	3
hsa-mir-26b-5p	THBS1, COL1A2, FN1	3
hsa-mir-29a-3p	COL4A1, ITGB1, COL1A1	3
hsa-mir-3609	THBS1, ITGB1, COL1A1	3

**Table 8:** Expression levels of five microRNAs in GC tissues and normal tissues.

miRNA Name	Cancer Abbreviation	T-Test P-value	T-Test FDR	Up regulated in:	Tumor Log2 Mean Expression	Normal Log2 Mean Expression
hsa-miR-29b-3p	**STAD**	**4.49e-02**	**7.67e-02**	**Tumor**	**9.76**	**9.23**
hsa-miR-29c-3p	STAD	3.68e-03	8.45e-03	Normal	11.35	12.10
hsa-let-7g-5p	**STAD**	**1.96e-02**	**3.75e-02**	**Tumor**	**9.36**	**9.02**
hsa-miR-218-5p	STAD	3.10e-03	7.36e-03	Normal	4.59	5.32
hsa-miR-29a-3p	**STAD**	**2.08e-02**	**3.96e-02**	**Tumor**	**13.33**	**12.82**

### 3.10 Using a public dataset to validate hub genes in GC

To confirm the reproducibility of the selected hub genes and to confirm the reliability of the integrated database a validation was performed using four random microarray datasets with the accession numbers GSE13911, GSE191275, GSE65801, and GSE174237, and 45687 (23385 upregulated and 22302 downregulated) DEGs were detected. A Venn diagram of these microarray datasets revealed that 621 DEGs were common between the datasets. After enrichment analysis, the results showed that extracellular matrix-receptor interactions (P < 0.05) were considerably enriched. During this process, we analyzed the differences in the expression of all genes related to normal-tumor tissues among the selected gastric tissue types with Match TCGA normal and GTEx data and found that all the hub genes, including ITGB1, COL1A1, COL1A2, COL4A1, FN1, COL6A3, THBS2, and THBS1, were significantly upregulated in GC tissues compared to normal tissues in this validated data series (Supplementary Fig. 5). Using bioinformatics analysis, various databases (GSE54129, GSE79973, GSE103236, GSE33651, GSE19826, and GSE118916) could provide highly reproducible results for underlying GC from intestinal etaplasia to adenocarcinoma.

## 4 Discussion

The association between histological and molecular alterations from IM to GC has not been fully characterized. Further investigations to pinpoint the potential alterations in the specific signaling pathways involved in the transition from IM to adenocarcinoma can predict both the progression and the potential for the early diagnosis of GC using high-throughput data.

In this study, six gene expression datasets for GC were employed, with 198 tumor samples and 82 nontumor samples, and one profile dataset for IM, with 14 IM samples and 15 healthy samples. Hence, a bioinformatics analysis was carried out, and a total of 7795 DEGs were identified. A Venn diagram revealed that 98 upregulated DEGs and 126 downregulated DEGs overlapped between a minimum of 4 datasets. For deep comprehensive bioinformatics, we performed functional enrichment analysis, protein–protein interaction (PPI) analysis, modular analysis, and identification of hub genes until we could predict potential therapeutic targets by examining genome-wide aberrations. We demonstrated that extracellular matrix-receptor interactions in GC and PPAR signaling pathway interactions in the IM might contribute to both the carcinogenesis and progression of underlying GC from the IM to adenocarcinoma. Therefore, ITGB1, COL1A1, COL1A2, COL4A1, FN1, COL6A3, THBS2, and THBS1 in GC and FABP1, APOC3, APOA1, HMGCS2, PPARA, and PCK1 in IM were the hub genes on which we focused.

Many analytical bioinformatics studies on GC have reported that the ECM is a key pathway of GC [35], and the role of the ECM in remodeling may have a profound impact on both the progression and prognosis of cancer [36]. However, to understand the molecular changes from IM to GC, the precancerous cascade must be considered. Clinical analysis of this study strongly indicated that ECM components (ITGB1, COL1A1, COL1A2, COL4A1, FN1, COL6A3, and THBS2) in GC were significantly correlated with overexpression and poor prognosis. Additionally, COL1A2, COL4A1, and COL6A3 had the highest mutation rates.

Recently, the role of ITGB1 (integrin β1, CD29) as a prognostic biomarker correlated with immunosuppression in GC has been demonstrated [37]. Importantly, previous studies suggested that ITGB1 may be an enhancer maintaining resistance to cancer chemotherapy [38]. Therefore, we focused our efforts on predicting the role of ITGB1 in GC progression and the signaling pathways involved. However, the current results demonstrated that ITGB1 could play a significant role in GC progression and might be a prognostic predictor and targeted therapy for GC.

Several collagen genes, COL1A1 and COL4A1, were previously reported to be overexpressed in GC and are closely related to overall survival in patients with GC and are considered risk factors for poor prognosis [39,40]. In the present study, COL1A1, COL1A2, COL4A1, and COL6A3 were found to be precisely expressed by collagen genes in human gastric lesions and could distinguish between malignant and premalignant lesions, identifying these genes as predictive biomarkers and/or therapeutic targets for GC [41].

FN1 (fibronectin 1) was one of the key genes consistently predicted by microarray and bioinformatics analyses of GC, and FN1 was also a hub gene, which is consistent with previous studies. High FN1 expression in GC cells was related to poor prognosis, and high FN1 expression in the ECM did not predict overall survival (OS) and was correlated with tumor progression [42]. Similarly, high expression of FN1 may be a positive tumor biomarker contributing to invasive breast cancer, pancreatic ductal adenocarcinoma (PDAC), and renal cell carcinoma (RCC) [[43], [44], [45]]. Tumor cell migration can be inhibited by blocking the FN1 signaling pathway [46]. Furthermore, another study reported that high expression of FN1, observed in cancer cells, is a tumor suppressor gene [47].

Thrombospondin-2 (THBS2), a member of the matricellular calcium-binding glycoprotein family, interacts with growth factors, cell receptor types, and the extracellular matrix (ECM) and contributes to cell proliferation, adhesion, and apoptosis [48]. THBS2 may also play a significant role in the detection of colon cancer [49], lung cancer [50], and GC [51]. However, the hub genes reported in this study are primarily involved in interactions between the ECM and receptors, digestion and absorption of proteins, focal adhesion, and the P13K-Akt signaling pathway, which are also activated in various cancers [52]. These results shed light on new therapeutic approaches for the clinical treatment of GC.

In the IM, GC is preceded by a cascade of precancerous lesions, known as the Correa cascade [36]. In relation to precancerous lesions and cancer development, the interplay of PPAR signaling pathways has been shown to be reverently represented in the IM. PPAR proteins show antitumor effects and are expressed in normal mucosa with IM adjacent to cancer [53]. This may represent a strategy to prevent IM from developing into cancer.

However, the present work revealed that FABP1, APOC3, APOA1, HMGCS2, PPARA, and PCK1 are important biomarkers that have received little attention in IM and may constitute a viable strategy for targeted GC therapy, providing some new insights for investigating the mechanism of GC generation.

Fatty acid binding protein (FABP1) might play an important role in fatty acid metabolism and is overexpressed in various cancers and promotes tumor angiogenesis and migration [54]. The expression of FABP1 correlates with the intensity of invasion and may guide the progression of GC and predict the risk of GC peritoneal metastasis [55]. Phosphoenolpyruvate carboxykinase 1 (PCK1) is known to be an enzyme that limits the rate of gluconeogenesis, but its role in both tumor metabolism and GC progression is unknown [56]. However, Kaplan‒Meier analysis revealed no association between PCK1 expression and prognosis. This may be because the Kaplan‒Meier analysis was performed on GC patients rather than on those with IM-GC.

Enrichment analysis of the IM samples indicated that the upregulated genes were mostly involved in digestive fat synthesis and secretion and absorption, cholesterol metabolism, vitamin digestion and absorption, bile secretion, glycolysis, and gluconeogenesis. All functions could be closely related to digestion and absorption. These findings suggest that changes in gastric digestive function and eating habits may be closely related to changes from healthy tissue to GIM.

A regulatory network of hub genes and their TFs was constructed to elucidate the molecular mechanisms of GC. In the present study, TP53, NR1H3, DMRT1, EZH2, JUN, AR, CLOCK, TCF4, SALL4, NFE2L2, SOX9, TET1, TP63, SOX2, ESR1, and PPARG were involved in the expression of the hub genes, where AR, TCF4, SALL4, and ESR1 were more important in this regulatory network. However, complex interactions between hub genes and TFS significantly contributed to the development of CC.

The translation of proteins or the inhibition of target mRNA cleavage can be regulated by miRNAs [57]. This study revealed that hsa-miR-29 may affect both the development and prognosis of GC by regulating hub genes. However, low expression of miR-29a in GC is associated with aggressive cancer biology and a decreased survival rate [58]. The transcript levels of miRNA-29 dramatically decline in several types of cancer [59,60].

miRNA-29 acts as an integrator and integral hub of key signaling pathways, such as nuclear factor-κB signaling, cell cycle, apoptosis, and epithelial mesenchymal transition (EMT) pathways [[61], [62], [63], [64]].

However, miR-29 can act via various upstream and target genes, as a commonly downregulated tumor suppressor gene, or as an oncogene in different kinds of cancer [65]. It is an important regulator in different types of human cancer. MiR-29a contributes to the development of GC, and miRNA-29 appears to have a significant effect on both the development and prognosis of GC [66,67]; however, due to its flexibility, the application of miR-29 as a biomarker and the development of miR-29a-based therapies for GC and its stages require further validation.

The identification of tumor suppressor genes was one of the major achievements of this study. DEGs between GC and IM were analyzed, showing that several genes (such as PTGR1, C1orf115, CRYL1, ALDOB, and SULT1B1) were present in both GC and IM; these genes were downregulated in GC and upregulated in IM. Notably, these genes may be tumor suppressor genes due to their dual roles in GC progression.

Prostaglandin reductase 1 (PTGR1) is known as the rate-limiting enzyme that contributes to the arachidonic acid pathway and is primarily involved in the inactivation of prostaglandins and several eicosanoids, such as leukotriene B4. Although its function in GC has not been studied, research has shown that PTGR1 is involved in the progression of many cancers [[68], [69], [70]]. It has also been suggested that PTGR1 may be involved in the proliferation of cancer cells and may be a potential target for treating cancer [68]. The sulfotransferases (SULTs), a family of enzymes that catalyze the sulfonation of various endogenous and exogenous substrates, include membrane-bound and cytosolic SULTs [71,72]. SULT1B1 is expressed at the highest levels in the intestine but is also present in moderate amounts in the liver, kidney, and white blood cells [73]. However, the known effect of SULT1B1 on GC has not been identified thus far and is likely important for the surveillance role of SULT1B1 in GC progression.

Furthermore, little is known about the roles of ALDOB, CRYL1, and C1orf115 in human cancer, especially GC, so future studies on these genes are needed. Further research and experiments on COL1A2, COL4A1, and COL6A3 in GC and PCK1 in IM and hsa-miR-29 will be particularly important. Our next research project will be to confirm the correlation between these core genes and GC.

Despite its strengths, the current study has limitations that warrant consideration; these limitations are outlined as following [1]: further experiments were needed to complement the bioinformatics analysis [2]; the basic features of the tumor, including gender, age, sample size, tumor grade and stage, and main misleading outcomes, were not considered [3]; a total of seven datasets were included, but no definitive results could be achieved; we used 12 GSE we should be data extraction from GEO and differentiated to up regulated and down regulated and gave Venn diagram for other downstream analyses performed on them, therefore we missed role some genes that work together.

## 5 Conclusion

This study identified new potential biomarkers and pathways in GC and IM that are important for underlying GC progression from IM to adenocarcinoma and revealed therapeutic targets in GC. It was demonstrated that ECM interactions in GC and PPAR signaling pathway interactions in IM may play key roles in the progression of underlying GC. COL1A2, COL4A1, and COL6A3 were significantly correlated with overexpression, poor prognosis, and the highest mutation rates in GC. FABP1, APOC3, APOA1, HMGCS2, PPARA, and PCK1 are important biomarkers that have received little attention in IM. Changes in gastric digestive function and eating habits may be closely related to changes from healthy tissue to IM. AR, TCF4, SALL4, and ESR1 were more important in the regulatory network of TF hub genes related to the expression of hub genes in GC. The development and prediction of GC via the regulation of hub genes may be affected by hsa-miR-29. PTGR1, C1orf115, CRYL1, ALDOB, and SULT1B1 may be tumor suppressor genes involved in GC progression. However, the primary conclusions of the analysis require further confirmation by a series of clinical experiments.

## Data availability statement

The datasets used during the current study, were deposited into the Gene Expression Omnibus database under accession number (GSE54129, GSE79973, GSE103236, GSE33651, GSE19826, GSE118916, GSE78523, GSE93415, GSE13911, GSE191275, GSE65801 and GSE174237 datasets) are available at the following URL https://www.ncbi.nlm.nih.gov/geo/.

## CRediT authorship contribution statement

**Mohammad Reza Eskandarion:** Writing – review & editing, Writing – original draft, Visualization, Validation, Supervision, Software, Resources, Project administration, Methodology, Investigation, Formal analysis, Data curation, Conceptualization. **Sharareh Eskandarieh:** Software, Methodology, Formal analysis. **Abbas Shakoori Farahani:** Writing – review & editing, Project administration, Methodology, Conceptualization. **Habibollah Mahmoodzadeh:** Writing – review & editing, Project administration, Conceptualization. **Farhad Shahi:** Writing – review & editing, Resources, Conceptualization. **Mohammad Ali Oghabian:** Writing – review & editing, Software, Methodology, Conceptualization. **Reza Shirkoohi:** Writing – review & editing, Writing – original draft, Validation, Supervision, Software, Project administration, Methodology, Funding acquisition, Conceptualization.

## Declaration of competing interest

The authors declare that they have no known competing financial interests or personal relationships that could have appeared to influence the work reported in this paper.
