# Supplementary material for: Prediction of novel biomarkers for gastric intestinal metaplasia and gastric adenocarcinoma using bioinformatics analysis
Source: Heliyon. 2024 Apr 25;10(9):e30253. doi: 10.1016/j.heliyon.2024.e30253 (PMC11088262; doi:10.1016/j.heliyon.2024.e30253)
Supplement: Multimedia component 1 [file mmc1.docx]

Supplementary Table 1. Gene Ontology of the DEGs in GC

| Expression | Gene Ontology | Term | P value | Genes |
| --- | --- | --- | --- | --- |
| Upregulated | **Biological process** | extracellular matrix organization (GO:0030198) | 7.68E-33 | ITGB1;COL18A1;SPARC;LAMA4;COL12A1;HTRA1;NID2;THBS1;LOXL1;SERPINH1;TIMP2;SPP1;COL10A1;TIMP1;LUM;FN1;BGN;COL1A1;VCAN;COL3A1;BMP1;COL1A2;COL5A1;COL4A1;ADAM12;COL5A2;MFAP2;COL8A1;COL6A3;LCP1;FBN1 |
|  |  | extracellular structure organization (GO:0043062) | 3.94E-26 | ITGB1;COL18A1;SPARC;LUM;LAMA4;FN1;BGN;NID2;THBS1;LOXL1;COL1A1;VCAN;COL3A1;COL1A2;COL5A1;COL4A1;ADAM12;COL5A2;MFAP2;SPP1;COL8A1;COL10A1;COL6A3;FBN1 |
|  |  | external encapsulating structure organization (GO:0045229) | 4.41E-26 | ITGB1;COL18A1;SPARC;LUM;LAMA4;FN1;BGN;NID2;THBS1;LOXL1;COL1A1;VCAN;COL3A1;COL1A2;COL5A1;COL4A1;ADAM12;COL5A2;MFAP2;SPP1;COL8A1;COL10A1;COL6A3;FBN1 |
|  |  | collagen fibril organization (GO:0030199) | 2.09E-19 | COL18A1;LUM;COL12A1;LOXL1;COL1A1;COL3A1;BMP1;COL1A2;COL5A1;COL4A1;COL5A2;SERPINH1;COL10A1;COL6A3;COL8A1 |
|  |  | supramolecular fiber organization (GO:0097435) | 1.22E-12 | COL18A1;LUM;ACTN1;COL12A1;LOXL1;CORO1C;COL1A1;COL3A1;BMP1;COL1A2;COL5A1;COL4A1;COL5A2;SERPINH1;COL8A1;COL10A1;COL6A3 |
|  | **Cellular Component** | platelet-derived growth factor binding (GO:0048407) | 1.15E-09 | COL1A1;COL3A1;COL1A2;COL5A1;COL4A1 |
|  |  | protease binding (GO:0002020) | 1.17E-07 | COL1A1;ITGB1;COL3A1;COL1A2;FAP;FN1;TIMP2;TIMP1 |
|  |  | metalloendopeptidase inhibitor activity (GO:0008191) | 3.99E-05 | RARRES1;TIMP2;TIMP1 |
|  |  | calcium ion binding (GO:0005509) | 0.001624 | SPARC;CDH3;CDH11;LCP1;THBS1;ASPN;FBN1 |
|  |  | endopeptidase inhibitor activity (GO:0004866) | 0.002473 | RARRES1;SERPINH1;TIMP2;TIMP1 |
|  | **Molecular Function** | collagen-containing extracellular matrix (GO:0062023) | 5.70E-34 | COL18A1;SPARC;LAMA4;COL12A1;HTRA1;AEBP1;THBS2;NID2;THBS1;LOXL1;LGALS1;SRPX2;SERPINH1;TIMP2;COL10A1;IGFBP7;HMCN1;CTHRC1;LUM;FN1;BGN;SULF1;ASPN;COL1A1;VCAN;COL3A1;COL1A2;COL5A1;COL4A1;COL5A2;MFAP2;COL8A1;COL6A3;FBN1 |
|  |  | endoplasmic reticulum lumen (GO:0005788) | 3.85E-19 | COL18A1;COL12A1;FN1;FSTL1;THBS1;COL1A1;VCAN;COL3A1;LGALS1;COL1A2;COL5A1;COL4A1;COL5A2;SERPINH1;SPP1;COL8A1;COL10A1;COL6A3;IGFBP7;TIMP1;FBN1 |
|  |  | intracellular organelle lumen (GO:0070013) | 3.35E-15 | COL18A1;SPARC;COL12A1;FSTL1;THBS1;GLS;LGALS1;ALDH1B1;SERPINH1;TIMP2;SPP1;COL10A1;IGFBP7;TIMP1;LUM;FN1;BGN;COL1A1;VCAN;COL3A1;COL1A2;COL5A1;COL4A1;COL5A2;COL8A1;COL6A3;FBN1 |
|  |  | platelet alpha granule (GO:0031091) | 5.30E-06 | SPARC;ACTN1;FN1;TIMP1;THBS2;THBS1 |
|  |  | platelet alpha granule lumen (GO:0031093) | 1.94E-05 | SPARC;ACTN1;FN1;TIMP1;THBS1 |

| Expression | Gene Ontology | Term | P value | Genes |
| --- | --- | --- | --- | --- |
| downregulated | **Biological process** | sodium ion homeostasis (GO:0055078) | 8.90E-09 | ATP4B;ATP4A;SCNN1G;SCNN1B;NEDD4L;SLC9A1 |
|  |  | cellular sodium ion homeostasis (GO:0006883) | 1.94E-06 | ATP4B;ATP4A;NEDD4L;SLC9A1 |
|  |  | monocarboxylic acid metabolic process (GO:0032787) | 3.46E-06 | CKMT2;CYP2C9;BTD;AKR1C1;ADH7;PTGR1;CYP2C18;CYP3A5 |
|  |  | cellular monovalent inorganic cation homeostasis (GO:0030004) | 6.73E-06 | ATP4B;ATP4A;NEDD4L;SLC9A1 |
|  |  | retinoid metabolic process (GO:0001523) | 2.54E-05 | AKR1B10;RDH12;AKR1C1;ADH7;CYP2C18;CYP3A5 |
|  | **Cellular Component** | oxidoreductase activity, acting on the CH-OH group of donors, NAD or NADP as acceptor (GO:0016616) | 3.97E-09 | CBR1;AKR1B10;HPGD;RDH12;AKR1C1;CRYL1;HADH;PTGR1;ADH7 |
|  |  | alcohol dehydrogenase (NADP+) activity (GO:0008106) | 3.35E-06 | ALDH3A1;AKR1B10;RDH12;AKR1C1 |
|  |  | aldo-keto reductase (NADP) activity (GO:0004033) | 2.84E-05 | ALDH3A1;AKR1B10;AKR1C1 |
|  |  | NAD+ binding (GO:0070403) | 6.67E-05 | HPGD;CRYL1;HADH |
|  |  | oxidoreductase activity, acting on NAD(P)H, quinone or similar compound as acceptor (GO:0016655) | 1.29E-04 | CBR1;NQO1;AKR1C1 |
|  | **Molecular Function** | integral component of plasma membrane (GO:0005887) | 7.54E-04 | ATP8B1;KCNJ15;TMPRSS2;KCNJ16;AQP4;LIFR;SSTR1;TSPAN12;SLC9A1;ATP4B;SCNN1G;ATP4A;SLC7A8;SCNN1B;ACKR4;SLC26A7;F2RL1;SLC26A9;GPRC5C;PROM2 |
|  |  | endoplasmic reticulum membrane (GO:0005789) | 0.001813 | GRAMD1C;CYP2C9;CYB5A;STS;MYRF;GDPD1;ELOVL6;GPAT3;CYP2C18;CYP3A5;SVIP;FOLR1 |
|  |  | cation-transporting ATPase complex (GO:0090533) | 0.00446 | ATP4B;SLC9A1 |
|  |  | sodium channel complex (GO:0034706) | 0.010745 | SCNN1G;SCNN1B |
|  |  | basolateral plasma membrane (GO:0016323) | 0.015366 | SLC7A8;SLC26A7;AQP4;FOLR1 |
