# Supplementary material for: Prediction of novel biomarkers for gastric intestinal metaplasia and gastric adenocarcinoma using bioinformatics analysis
Source: Heliyon. 2024 Apr 25;10(9):e30253. doi: 10.1016/j.heliyon.2024.e30253 (PMC11088262; doi:10.1016/j.heliyon.2024.e30253)
Supplement: Multimedia component 2 [file mmc2.docx]

Supplementary Table 2. Gene Ontology of DEGs in IM

| Expression | Gene Ontology | | Term | P value | Genes |
| --- | --- | --- | --- | --- | --- |
| Upregulated | **Biological process** | | heme catabolic process (GO:0042167) | 4.05E-08 | FABP1;ABCC2;UGT1A1;SLCO2B1;GSTA1;HMOX1;ABCG2 |
|  |  |  | porphyrin-containing compound catabolic process (GO:0006787) | 4.05E-08 | FABP1;ABCC2;UGT1A1;SLCO2B1;GSTA1;HMOX1;ABCG2 |
|  |  |  | lipid transport (GO:0006869) | 4.94E-08 | ABCG8;ABCG5;PRELID1;STARD5;ABCC2;MTTP;NR1H4;APOA1;APOA4;SLC51A;SLC51B;GLTPD2;FABP2;CEACAM1;SLCO2B1 |
|  |  |  | organic hydroxy compound transport (GO:0015850) | 3.34E-07 | ABCG8;ABCG5;CEACAM1;ABCC2;STARD5;SLCO2B1;NR1H4;SLC51A;SLC51B |
|  |  |  | organic acid transport (GO:0015849) | 7.56E-07 | SLC46A1;SLC38A1;SLC6A19;STARD5;ABCC2;NR1H4;SLC3A1;SLC51A;SLC51B;CEACAM1;SLC7A9;SLCO2B1;ABCG2 |
|  | **Cellular Component** | | basolateral plasma membrane (GO:0016323) | 3.75E-09 | SLC46A1;SLC38A1;TFRC;ANXA2;MTTP;ATP1B3;ANK3;SLC51A;ATP1A1;SLC51B;CLDN1;SLC4A4;SLC4A7;ATP7B;HEPH;EPCAM;MYO1A;CDH17;CD44 |
|  |  |  | brush border membrane (GO:0031526) | 1.08E-08 | ACE2;SLC6A19;SLC7A9;CDHR2;ITLN1;SLC3A1;CYBRD1;CDHR5;SLC5A1;ABCG2 |
|  |  |  | cell projection membrane (GO:0031253) | 1.22E-05 | ACE2;SLC9A3R1;SLC6A19;SLC7A9;CDHR2;ITLN1;SLC3A1;CYBRD1;CDHR5;SLC5A1;ABCG2 |
|  |  |  | intracellular organelle lumen (GO:0070013) | 1.61E-05 | CDA;APP;SERPINA1;ECHS1;OAT;ACAA2;MTTP;CTSZ;MSLN;GLS;HSPD1;MUC2;C1QBP;MUC12;MUC13;HMGCS2;APOB;MUC4;PGM1;CTSC;IDH3A;PDK1;PCK2;DEFA6;MUC17;DEFA5;APOA1;APOA4;GCG;OLFM4;MUC3A;GIP;SOD1;ACE2;CPS1;ETHE1;MGAT4A;SUCLG1;ZG16;SLC27A2;CES2 |
|  |  |  | microvillus (GO:0005902) | 5.94E-05 | VIL1;SLC9A3R1;MYO1A;CDHR2;MYO7B;CDHR5;ESPN;CD44 |
|  | **Molecular Function** | | cholesterol transfer activity (GO:0120020) | 1.08E-07 | ABCG8;ABCG5;STARD5;MTTP;APOA1;APOA4;APOB |
|  |  |  | sterol transfer activity (GO:0120015) | 1.67E-07 | ABCG8;ABCG5;STARD5;MTTP;APOA1;APOA4;APOB |
|  |  |  | organic anion transmembrane transporter activity (GO:0008514) | 9.50E-06 | SLC46A1;SLC38A1;ABCC2;SLC35A3;SLC1A1;SLC3A1;SLC9A3R1;SLC25A15;SLC6A8;SLC7A9;SLCO2B1;SLC26A3;CFTR;ABCG2 |
|  |  |  | solute:sodium symporter activity (GO:0015370) | 6.58E-05 | SLC4A7;SLC5A9;SLC20A2;SLC5A1;SLC4A4;SLC17A4 |
|  |  |  | transition metal ion transmembrane transporter activity (GO:0046915) | 6.58E-05 | SLC25A37;ATP7B;SLC30A4;SLC39A5;SLC30A10;SLC39A4 |
| downregulated | | **Biological process** | neurotransmitter uptake (GO:0001504) | 1.13E-05 | SNAP25;SLC29A1;GLUL |
|  |  |  | regulation of sprouting angiogenesis (GO:1903670) | 2.65E-04 | FUT1;GLUL;KLF2 |
|  |  |  | fucose catabolic process (GO:0019317) | 5.04E-04 | FUT9;FUT1 |
|  |  |  | L-fucose catabolic process (GO:0042355) | 5.04E-04 | FUT9;FUT1 |
|  |  |  | L-fucose metabolic process (GO:0042354) | 5.04E-04 | FUT9;FUT1 |
|  |  | **Cellular Component** | vesicle (GO:0031982) | 0.010804 | SNAP25;FGA;MUC1;SLC5A5 |
|  |  |  | cytoplasmic side of lysosomal membrane (GO:0098574) | 0.018858 | EEF1A2 |
|  |  |  | extracellular membrane-bounded organelle (GO:0065010) | 0.019224 | FGA;SLC5A5 |
|  |  |  | extracellular vesicle (GO:1903561) | 0.021204 | FGA;SLC5A5 |
|  |  |  | endoplasmic reticulum lumen (GO:0005788) | 0.023266 | FGA;TSPAN5;CHGB;CES1 |
|  |  | **Molecular Function** | fucosyltransferase activity (GO:0008417) | 9.18E-04 | FUT9;FUT1 |
|  |  |  | hormone activity (GO:0005179) | 0.00327 | SST;GAST;CHGB |
|  |  |  | endopeptidase inhibitor activity (GO:0004866) | 0.009602 | CSTA;PCSK1N;SPINK1 |
|  |  |  | peptidase inhibitor activity (GO:0030414) | 0.010124 | PCSK1N;SPINK1 |
|  |  |  | hexosyltransferase activity (GO:0016758) | 0.010534 | FUT9;B4GALNT3;FUT1 |
