# Supplementary material for: Prediction of novel biomarkers for gastric intestinal metaplasia and gastric adenocarcinoma using bioinformatics analysis
Source: Heliyon. 2024 Apr 25;10(9):e30253. doi: 10.1016/j.heliyon.2024.e30253 (PMC11088262; doi:10.1016/j.heliyon.2024.e30253)
Supplement: Multimedia component 3 [file mmc3.docx]

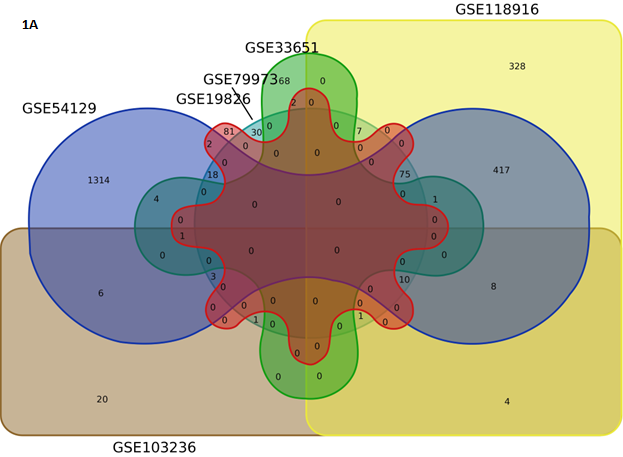


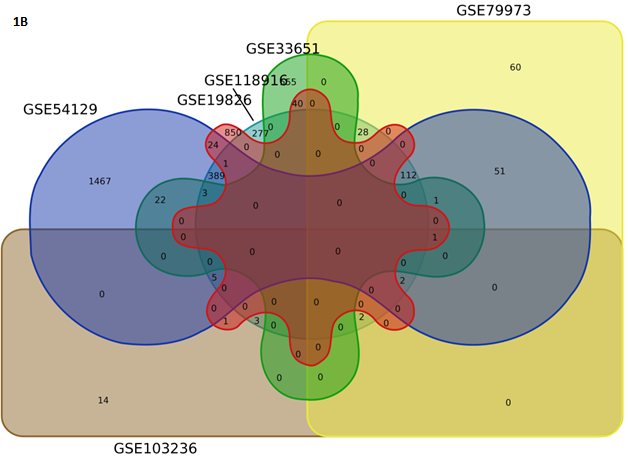


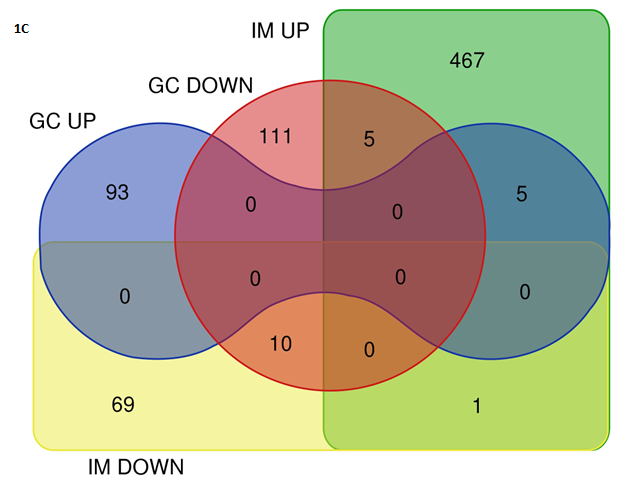


supplementary Figure 1. Filtering of DEGs in GEO database. **A**. Venn diagram of the up -regulated DEGs in the GSE54129, GSE79973, GSE103236, GSE33651, GSE19826, and GSE118916. **B**. Venn diagram of the down- regulated DEGs in the GSE54129, GSE79973, GSE103236, GSE33651, GSE19826, and GSE118916. **C**. Venn diagram of up / down regulated in GC compared to that of up/down regulated in IM.
