# Supplementary material for: Prediction of novel biomarkers for gastric intestinal metaplasia and gastric adenocarcinoma using bioinformatics analysis
Source: Heliyon. 2024 Apr 25;10(9):e30253. doi: 10.1016/j.heliyon.2024.e30253 (PMC11088262; doi:10.1016/j.heliyon.2024.e30253)
Supplement: Multimedia component 4 [file mmc4.docx]

2A


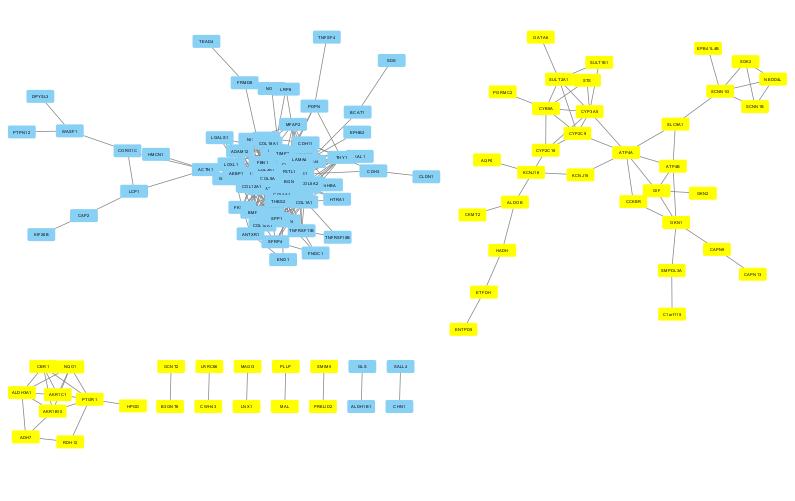
2B


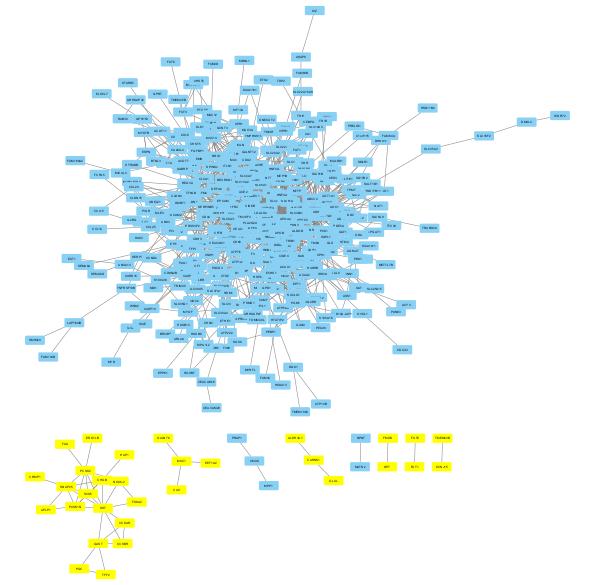


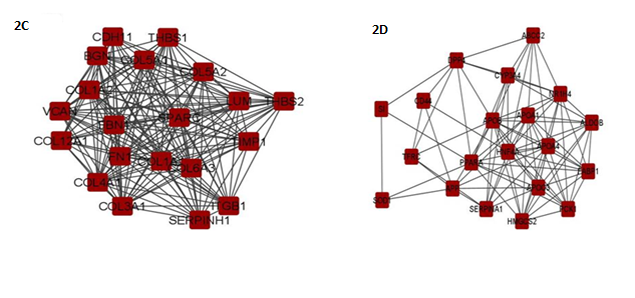


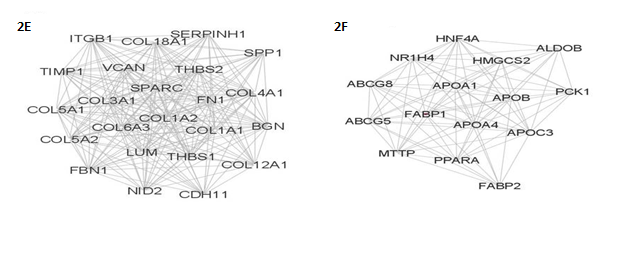


Supplementary Figure 2: An analysis of PPI network: (a) A diagram showing PPI network of shared differentially genes in GC; (b) A diagram representing PPI network of shared differentially genes in IM (c) core targets observed in GC; (d) core targets found in IM (e) core subnets with a score > 5 based on MCODE in gastric cancer (f) core network of subnets showing a score > 5 according to MCODE in IM.
