# Supplementary material for: Prediction of novel biomarkers for gastric intestinal metaplasia and gastric adenocarcinoma using bioinformatics analysis
Source: Heliyon. 2024 Apr 25;10(9):e30253. doi: 10.1016/j.heliyon.2024.e30253 (PMC11088262; doi:10.1016/j.heliyon.2024.e30253)
Supplement: Multimedia component 5 [file mmc5.docx]

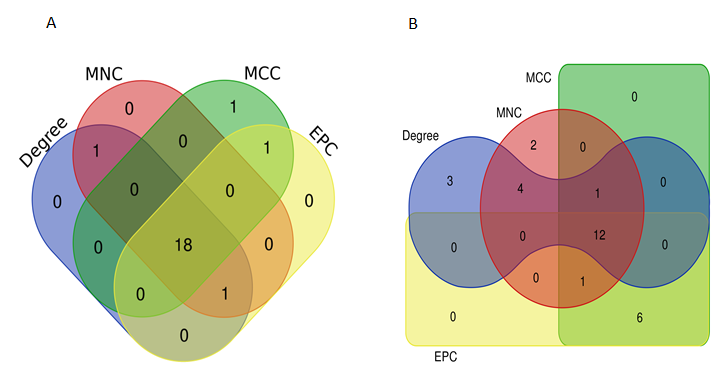


Supplementary Figure 3. The Venn diagram for the top 20 genes identified with degree, MNC, MCC, and EPC methods was used that 18 hub genes in gastric cancer (A) and 12 hub genes in IM (B) were select.
