# Supplementary material for: Prediction of novel biomarkers for gastric intestinal metaplasia and gastric adenocarcinoma using bioinformatics analysis
Source: Heliyon. 2024 Apr 25;10(9):e30253. doi: 10.1016/j.heliyon.2024.e30253 (PMC11088262; doi:10.1016/j.heliyon.2024.e30253)
Supplement: Multimedia component 6 [file mmc6.docx]

**Supplementary Figure 4A*)***

*
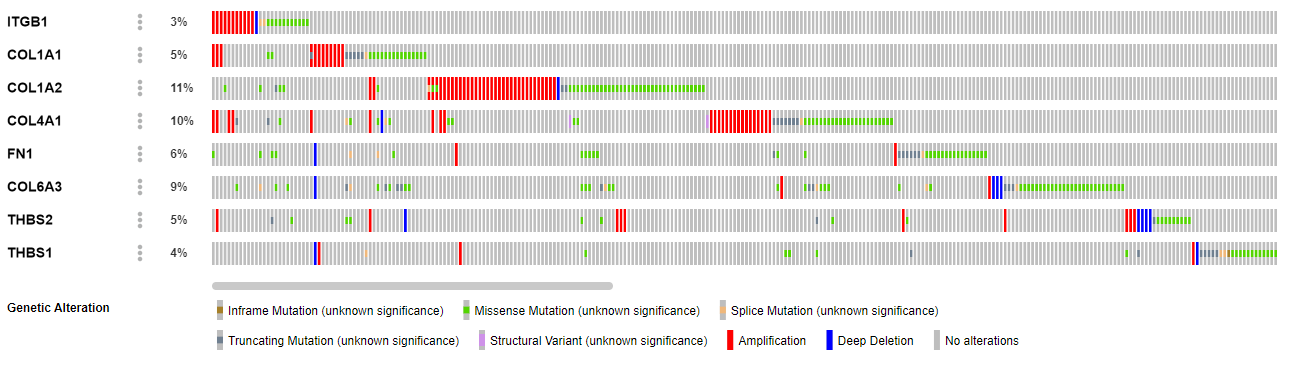
*

**Supplementary Figure 4B*)***

*
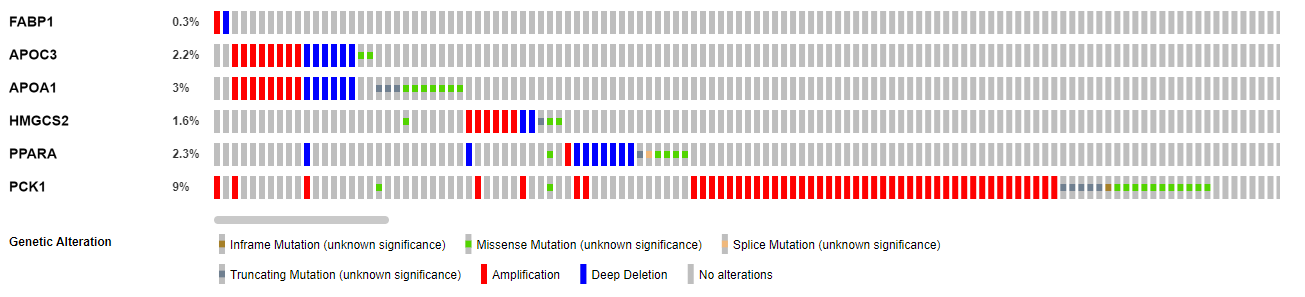
*

**Supplementary Figure 4** ***.* Genetic mutation analysis of hub genes in GC and IM**. 4A) The mutation rate is 11% for COL1A2; 10% for COL4A1 and 9% for COL6A3; 6% for FN1, 5% for COL1A1 and THBS2;4% for THBS1 and 3% for ITGB1 in GC. 4B) The mutation rate is 9% for PCK1, 3% for APOA1 and less than 3% for APOC3, PPARA, HMGCS2, FABP1.
