# Supplementary material for: Prediction of novel biomarkers for gastric intestinal metaplasia and gastric adenocarcinoma using bioinformatics analysis
Source: Heliyon. 2024 Apr 25;10(9):e30253. doi: 10.1016/j.heliyon.2024.e30253 (PMC11088262; doi:10.1016/j.heliyon.2024.e30253)
Supplement: Multimedia component 7 [file mmc7.docx]

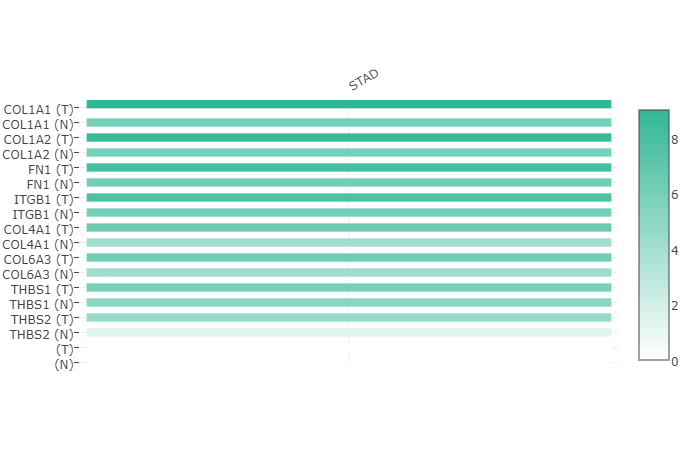


Supplementary figure 5. Validation of hub genes using GSE13911, GSE191275, GSE65801, GSE174237 public data series. All 8 hub genes selected including ITGB1, COL1A1, COL1A2, COL4A1, FN1, COL6A3, THBS2 and THBS1 were significantly upregulated in GC tissues compared to normal tissues in this validated data series. T: Tumor samples and N: Normal samples
